# Atopic Dermatitis and Ulcerative Colitis Successfully Treated with Upadacitinib

**DOI:** 10.3390/medicina59030542

**Published:** 2023-03-10

**Authors:** Teresa Grieco, Martina Caviglia, Giuseppina Cusano, Alvise Sernicola, Camilla Chello, Ester Del Duca, Carmen Cantisani, Alberto Taliano, Nicolò Sini, Gianluca Ianiro, Giovanni Pellacani

**Affiliations:** 1 Dermatology Unit, Department of Clinical Internal Anesthesiological and Cardiovascular Sciences, “Sapienza” University of Rome, 00161 Rome, Italy; 2 Department of Translational and Precision Medicine, “Sapienza” University of Rome, 00161 Rome, Italy; 3 Digestive Disease Center, Fondazione Policlinico Universitario Gemelli IRCCS, Università Cattolica del Sacro Cuore, 00168 Rome, Italy

**Keywords:** upadacitinib, atopic dermatitis, ulcerative colitis, patient selection, Janus kinase

## Abstract

*Background and Objectives*: JAK inhibitors entered current clinical practice as treatment for several immune-related diseases and, recently, for atopic dermatitis. These drugs target the Janus Kinase intracellular cascade, rendering them suitable for treating both Th1 and Th2 immune-mediated responses. *Materials and Methods*: We report the case of a 36-year-old male patient presenting an overlap of ulcerative colitis, a Th1-related disease, and atopic dermatitis, a Th2-mediated condition. Treatment with upadacitinib was initiated, and laboratory and instrumental follow-ups were carried out for 8 months. *Results*: The complete and persistent clinical remission of both conditions was observed at a low dose of 15 mg of upadacitinib, even though ulcerative colitis guidelines usually recommend a dosage of 45 mg. No serious adverse responses to therapy were reported. *Conclusions*: Upadacitinib may be the most suitable management strategy in subjects with coexisting severe conditions mediated by Th1 inflammation, such as ulcerative colitis, and by Th2 cytokines, such as atopic dermatitis.

## 1. Introduction

Janus kinase inhibitors (JAKi) are a class of oral medications belonging to the group of “small molecules”. These new drugs have been developed over the past decade and are classified as pan-JAKi or selective JAKi based on the spectrum of the Janus kinase members targeted. The Janus Kinase–Signal Transducer and Activator of Transcription (JAK-STAT) pathway is a key driver of inflammation-transducing proinflammatory and proliferative cytokine signals to the nucleus [1], and it plays a crucial role in the pathogenesis of immune-mediated inflammatory disorders (IMIDs), including psoriasis, atopic dermatitis (AD), autoimmune disorders (AID), inflammatory bowel diseases (IBD), and even cancer [1]. JAKis act by inhibiting JAK-STAT signaling in the cytosol; thus, they represent promising alternatives to parenteral biological therapy and disease-modifying antirheumatic drugs (DMARDs) in IMIDs [2]. Abrocitinib and upadacitinib, new generation JAK1-selective inhibitors, are consistently effective therapies in adults and adolescents with AD. In 2019, upadacitinib received approval by the US Food and Drug Administration (FDA) and by the European Medicines Agency (EMA) for the treatment of rheumatoid arthritis, ankylosing spondylitis, psoriatic arthritis, and severe AD, offering the advantage of oral administration rather than the injective route for the delivery of monoclonal antibodies. Pivotal upadacitinib trials (Measure Up 1, Measure Up 2 and AD Up) proved that this molecule is an optimal option for short-term AD treatment, especially with respect to improving itch according to the NRS for pruritus (3). In the same year, this drug was approved for both the induction phase and maintenance therapy of moderate-to-severe ulcerative colitis (UC) by the FDA and, in July 2023, in European countries as well [3].

We report the case of a subject suffering from both AD and UC who was treated with low-dose-upadacitinib, for which the efficacy and tolerability of this drug in the treatment of both conditions were demonstrated.

## 2. Materials and Methods

In our AD outpatient unit at “Sapienza” University of Rome, we performed total skin examination and a full laboratory protocol for allergy and autoimmune diseases, including blood test, specific IgE test for respiratory and food allergens, skin prick tests, and patch test. The results of laboratory examinations are reported in Table 1. The Eczema Area and Severity Index (EASI), Investigator Global Assessment (IGA), Pruritus Numerical Rating Scale (NRS), and Dermatology Life Quality Index (DLQI) were also employed as clinical scoring systems in accordance with current AD guidelines [4]. In addition, the patient is currently undergoing regular visits at the Gastroenterology Unit of Policlinico Gemelli in Rome for the management and follow-up of UC in accordance with the latest American College of Gastroenterology’s (ACG) clinical guidelines [5]. Gut microbiota analysis was also performed at the same center.

Images of cutaneous lesions were captured during each visit. The patient analyzed in this manuscript provided their written informed consent for the publication of their case details and photographs. The study was conducted according to the guidelines of the Declaration of Helsinki. Ethical review and approval have been waived for this study as they are not required by our Institutional Ethics Committee (Comitato Etico–Azienda Policlinico Umberto I) for reports of individual cases.

## 3. Case Presentation

**Figure 1:**
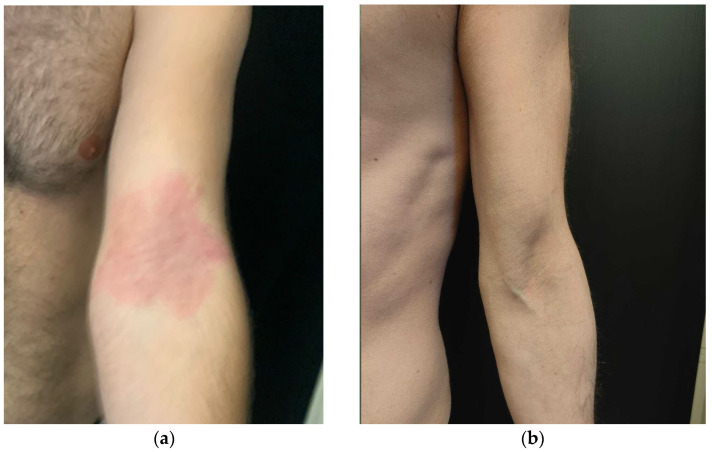
Atopic dermatitis. Clinical examination at the time of presentation, showing severe flexural involvement with erythema, papulation, and lichenification (**a**); then, after one month of therapy, the upadacitinib treatment’s induction of clear skin can be seen (**b**).

**Figure 2:**
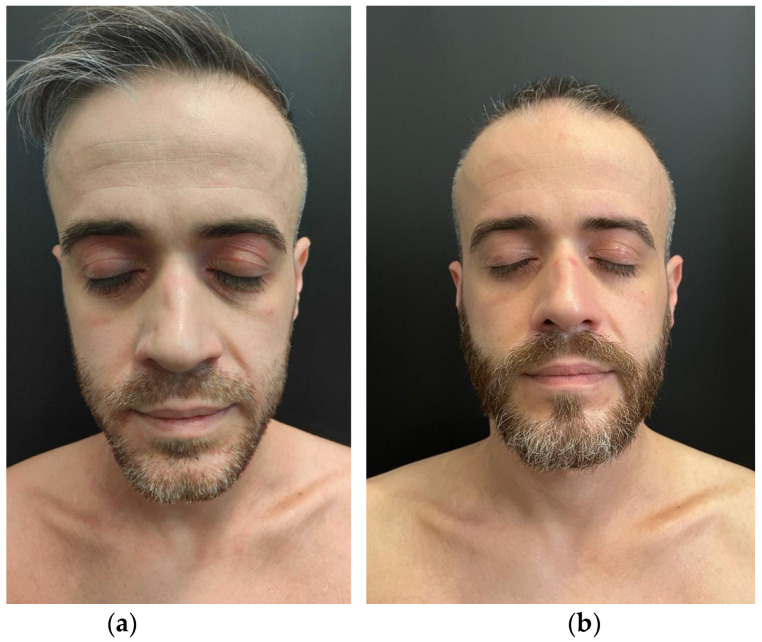
Atopic dermatitis. Clinical examination at the time of presentation showing eyelid edema, erythema, and lichenification together with peri-orbital darkening and facial pallor (**a**); then, after one month of therapy with upadacitinib, minimal signs of atopic dermatitis can be seen (**b**).

A 36-year-old Caucasian male suffering from severe generalized AD that had been broadly impacting on his quality of life came to our attention in 2021. The patient’s personal history revealed UC treated with mesalamine and beclomethasone since 2020. The baseline severity of AD was measured as follows: EASI 30, IGA 4, Pruritus-NRS 10, DLQI 14 (Figure 1a and Figure 2a). The patient was non-responsive to topical therapies, such as high-potency corticosteroids and tacrolimus ointment, and to systemic therapies, such as methylprednisolone and cyclosporine, which were administered at 4 mg/kg/day for more than 6 months. Therefore, the patient was considered eligible for dupilumab therapy at the scheduled dosing of 600 mg sc followed by 300 mg sc every 2 weeks. After 6 months of follow-up, the patient was considered a “low responder” to this biological drug as well.

During this period, the patient showed exacerbation of UC with rectorrhagia, abdominal pain, diarrhea (>3 times a day), an increase in quantitative fecal calprotectin (218 mg/g), serum inflammatory markers, eosinophilia (1 × 10^3^ cells/uL), and ASCA and ANCA positivity (Table 1). In May 2022, with the aim of more effectively managing both conditions, that is, AD and UC, the patient started oral upadacitinib at a dose of 15 mg once daily.

After one month of therapy, we documented a dramatic clinical response in terms of of AD with EASI <1, NRS-Itch 2, and DLQI 2 (Figure 1b and Figure 2b). Concurrently, we observed a complete remission of symptoms related to UC. The laboratory follow-up for UC was uneventful, showing quantitative fecal calprotectin of 120 mg/g (ULN = 120 mg/g) in September, with further reduction below normal value in November 2022.

Substantial improvement of the patient’s inflammatory parameters (Table 1) and endoscopic evaluation confirmed the remission of UC. No gastrointestinal symptomatology such as diarrhea or abdominal pain were referred. No serious adverse effects related to JAKi were reported after a follow up of 8 months; to improve dyslipidemia, which is possibly related to the drug, the patient started treatment with 10 mg of simvastatin daily.

## 4. Discussion

AD is a common immune-mediated skin disorder characterized by eczematous lesions, pruritus, and a chronic, relapsing behavior. The prevalence of AD among adult subjects has been estimated to range from 0.3% to 14.3% across studies, and the disease is being increasingly recognized among elderly subjects, with a prevalence of at least 1–3%. AD and its associated comorbidities have a deep impact on patients’ quality of life and the management of this disease requires a global approach tailored to the specific comorbidities and concerns of each subject. The pharmacological management of moderate-to-severe AD, when the disease is unresponsive to topical treatments or phototherapy, requires the use of systemic treatments [6].

The availability of a treatment option with sufficient efficacy and safety in the long term has been a sorely unmet need in the therapeutic management of AD until the recent introduction of dupilumab. Dupilumab, a fully human IL-4Ralpha blocker, was the first biologic drug approved for the treatment of moderate-to-severe AD, asthma, and chronic rhinosinusitis with nasal polyps. This monoclonal antibody operates through the inhibition of the IL-4 and IL-13 signaling pathways, which are key for type 2 inflammation, by targeting the IL-4Ralpha/IL-4RgammaC receptor dimer and IL-4Ralpha/IL-13Ralpha1 receptor dimer, respectively. Dupilumab is currently approved for the treatment of moderate-to-severe AD by the FDA for patients aged 6 years and above who are “not adequately controlled with topical therapies or when those therapies are not advisable” and by the EMA for patients aged 6 years or above who are eligible for systemic therapy. The current Italian guidelines for the therapy of AD, adapting the latest consensus-based European Dermatology Forum (EDF) guidelines for the treatment of AD, have implemented these indications in the use of biologic agents [6].

Parallel to the recent treatment advancements, our understanding of the pathogenesis of AD is rapidly evolving: following the identification of Th2 skewed immunity, altered skin barrier, and abnormal keratinocyte polarization, the role of cutaneous dysbiosis has been recently recognized to contribute to inefficient barrier function and susceptibility to infection [7]. In a study by Hammond et al., the bacterial and fungal microbiomes were assessed at skin sites at which AD had manifested and compared with those of subjects with non-atopic dermatitis [8]. The results showed an increase in *Staphilococcus aureus*, *Lactococcus*, and *Alternaria* in samples from AD-affected skin. *Alternaria* is an established pathogen linked to asthma and AD and its antigens have been hypothesized to play a role in the development of allergies; for instance, filaggrin mutation and sensitization with *Alternaria* were shown to enhance sensitization to food and, possibly, respiratory allergens [9]. Moreover, mixed biofilms formed by *Staphilococcus* and *Alternaria* are capable of worsening AD by negatively affecting barrier function and modulating the expression of pro-inflammatory cytokines such as thymic stromal lymphopoietin (TSLP) [8]. These results suggest that in AD subjects with skin barrier disfunction, the microbiome has a prominent role in transcutaneous sensitization. Accordingly, cutaneous dysbiosis may also be able to trigger and sustain intestinal dysbiosis and affect the development of IBD.

Finally, in subjects with cutaneous dysbiosis, immunomodulatory agents including monoclonal antibodies and small-molecule inhibitors would be preferable treatments together with future approaches targeting the microbiome with respect to AD [10].

AD is commonly accompanied by additional atopic disorders of the mucous membranes that constitute the atopic march affecting the airway mucosae (with manifestations such as asthma and rhinitis) and the intestinal epithelium, where atopy may act as a trigger for IBD [11]. In this regard, UC has been described as a Th2-dominant disease, and associations with AD are often reported [12].

Nonetheless, the literature has shown that the modulation of IL-13 signaling is incapable of improving UC [13]. Other authors have proposed that dupilumab targets the IL-4 pathway in IBDs, where it may have a protective role [14], but it did not prove beneficial in our case.

AD, like other allergic conditions such as food allergy, asthma, and rhino-conjunctivitis, is a typical Th2 inflammatory disease mediated by the key cytokines IL-4 and IL-13. This pathway is distinct from so-called Th1/Th17 immunity, which is mediated by IFN-gamma, TNF, and IL-17 and associated with diseases such as psoriasis, seronegative spondyloarthropathy, granulomatous inflammation in sarcoidosis, and other conditions [15]. Despite the initial proposal of distinctive Th cell lineages, it is currently an established fact that there is an overlap between the various lineages and their forms of cytokine production [16]. In this context, the dupilumab-mediated blockage of Th2 responses may promote an imbalance toward the development of Th1- and Th17-related disorders. It has been demonstrated that IL-4 and IL-13 downregulate both IL-23 and IL-17; therefore, dupilumab may promote paradoxical reactions mediated by the activation of the IL-23/IL-17 cytokine pathway [15].

The JAK family members are key drivers of the signaling of multiple proinflammatory cytokines that play a crucial role in AD pathogenesis, i.e., cytokines belonging to the Th2/Th22 pathway, such as IL-4, IL-13, IL-31, TSLP, IL-22, and Th1/Th17 cytokines, of which the foremost include IFN-gamma and IL-17. The same behavior occurs in UC, where the JAK molecules mediate the activity of IL-6, IL-12, IL-23, and IFN-gamma, which are all Th1/17 family members [17].

Upadacitinib is a small JAK1-selective molecular inhibitor that acts by blocking the key interleukins of adaptive immunity (IL-2, IL-4, IL-7, IL-9, IL-13, IL-15, and IL-21) and several proinflammatory cytokines such as IL-6 and the type-I interferons [18]. In UC, a Th2-like differentiation process leads to the expansion of Natural Killer T (NKT) cells, especially IL-13–producing NKT cells. These cytokine patterns result in the activation of Th1/Th17–Th2 pathways with the involvement of TNF-alfa, IL-1beta, and IL-6 [19,20]. This evidence explains why JAK1-selective inhibitors may block the key cytokine pathways involved in the pathogenesis of both inflammatory conditions, with AD affecting the skin and UC involving the gut.

## 5. Conclusions

The successful use of upadacitinib in a patient with an overlap of AD and UC was supported by phase III randomized controlled trials (RCTs) that have shown the efficacy of upadacitinib with respect to achieving clinical and endoscopic remission in patients with moderate-to-severe UC compared to previous biologic therapies. Moreover, the successful response to the 15 mg low dosage of JAKi administered to our patient led a noteworthy result when compared to the recommended regimen for UC (45 mg induction and 30 mg maintenance therapy).

A complete remission of symptomatology and the normalization of laboratory tests were observed in our patient in a very short time, thus drawing attention to the remarkable benefits of anti-JAK1 small-molecule therapy in dermatology. Therefore, upadacitinib may be the most suitable management strategy among subjects with coexisting severe conditions mediated by Th1 inflammation, such as UC, and by Th2 cytokines, such as AD.

## Figures and Tables

**Table 1:** Results of laboratory analysis performed in April 2021, before initiation of treatment with dupilumab; in March 2022, before switching to upadacitinib; and in September 2022 and January 2023, after 4 and 8 months of therapy with upadacitinib, respectively. Values outside normal laboratory reference ranges are marked with *.

Analysis	Unit	12 April 2021	18 March 2022	8 September 2022	2 January 2023
Hemoglobin	g/dL	15.2	15.6	14	14.5
Red blood cells	cells/uL	5.27 × 10^6^	5.40 × 10^6^	4.91 × 10^6^	4.84 × 10^6^
Eosinophils	cells/uL (%)	600 (8.9%) *	600 (9.2%) *	500	300 (4.2%)
Total bilirubin	mg/dL	N/A	N/A	1.21	1.48 *
Direct bilirubin	mg/dL	N/A	N/A	0.36	0.48
AST	IU/L	24	21	21	31
ALT	IU/L	27	20 L	29	28
GGT	IU/L	N/A	20	31	29
Total cholesterol	mg/dL	N/A	262 *	254 *	222 *
HDL cholesterol	mg/dL	N/A	88	N/A	89
CPK	IU/L	N/A	N/A	N/A	212 *
Total IgE	IU/mL	839 *	417 *	N/A	986 *
ESR	mm/h	3 mm	4 mm	4 mm	4 mm
CRP	mg/dL	0.08	<0.06	<0.06	<0.06
ASCA	titer	N/A	1:80 *	N/A	<1:80
MPO-ANCA	IU/mL	N/A	<3.2	N/A	<3.2
PR3-ANCA	IU/mL	N/A	87 *	N/A	9
Fecal calprotectin	mg/g	Positive *	218 *	120	<120
FOBT		Positive *	N/A	N/A	negative

Abbreviations: ALT, alanine transaminase; ASCA, anti-Saccharomyces cerevisiae antibodies; AST, aspartate transaminase; CPK, creatine phosphokinase; CRP, C-reactive protein; ESR, erythrocyte sedimentation rate; FOBT, fecal occult blood test; GGT, gamma-glutamyl transferase; HDL, high-density lipoprotein; IgE, immunoglobulin E; MPO-ANCA, anti-myeloperoxidase anti-neutrophil cytoplasmic antibodies; N/A, not available; PR3-ANCA, anti-proteinase 3 anti-neutrophil cytoplasmic antibodies.

## Data Availability

The data presented in this study are available from the corresponding author upon request. The data are not publicly available due to privacy restrictions.
